# Exhaled carbon monoxide: a non-invasive biomarker of short-term exposure to outdoor air pollution

**DOI:** 10.1186/s12889-017-4243-6

**Published:** 2017-04-17

**Authors:** Herve Lawin, Lucie Ayi Fanou, Vikkey Hinson, Jacqueline Wanjiku, N. Kingsley Ukwaja, Stephen B Gordon, Benjamin Fayomi, John R Balmes, Parfait Houngbegnon, Euripide Avokpaho, Ambaliou Sanni

**Affiliations:** 1 Unit of Teaching and Research in Occupational and Environmental Health, Department of Public Health, Faculty of Health Sciences, University of Abomey-Calavi, Cotonou, Benin; 2 Institut Regional de Santé Publique, University of Abomey Calavi, Cotonou, Benin; 3 Laboratoire de Biochimie et de Biologie Moléculaire, FAST/UAC, Cotonou, Benin; 4 Department of Internal Medicine, Egerton University, Njoro, Kenya; 5 Department of Internal Medicine, Federal Teaching Hospital, Abakaliki, Ebonyi State Nigeria; 6 Liverpool School of Tropical Medicine, Liverpool, UK; 7 Division of Environmental Health Sciences, School of Public Health, University of California, Berkeley, USA

**Keywords:** Carbon monoxide, Air pollution, Biomarker

## Abstract

**Background:**

In urban settings of Africa with rapidly increasing population, traffic-related air pollution is a major contributor to outdoor air pollution (OAP). Although OAP has been identified as a leading cause of global morbidity and mortality, there is however, lack of a simple biomarker to assess levels of exposure to OAP in resource-poor settings. This study evaluated the role of exhaled carbon monoxide (exhCO) as a potential biomarker of exposure to ambient carbon monoxide (ambCO) from OAP.

**Methods:**

This was a descriptive study conducted among male commercial motorcycle riders in Cotonou – the economic capital of Benin. The participants’ AmbCO was measured using a portable carbon monoxide (CO) data logger for 8 h during the period of their shift. ExhCO was measured just before and immediately after their shift (8-h) Participants were asked not to cook or to smoke during the day of the measurements. Linear regression analysis was used to assess the association between ambCO and exhCO for the last 2, 4 and 6 h of their shift.

**Results:**

Of 170 participants who completed the study, their mean ± SD age was 42.2 ± 8.4 years, and their mean ± SD daily income was 7.3 ± 2.7$. Also, 95% of the participants’ used solid fuels for cooking and only 2% had ever smoked. Average exhCO increased by 5.1 ppm at the end of the shift (*p* = 0.004). Post-shift exhCO was significantly associated to ambCO, this association was strongest for the last 2 h of OAP exposure before exhCO measurement (β = 0.34, *p* < 0.001).

**Conclusion:**

ExhCO level was associated with recent exposure to ambCO from OAP with measurable increase after 8 h of exposure. These findings suggest that ExhCO may be a potential biomarker of short-term exposure to OAP.

**Electronic supplementary material:**

The online version of this article (doi:10.1186/s12889-017-4243-6) contains supplementary material, which is available to authorized users.

## Background

Outdoor air pollution (OAP) especially from vehicular traffic is of growing importance since it occurs largely in urban areas expected to constitute 66% of the world population by 2050 [1]. According to the United Nations [1], much of the expected urban growth will take place in low and middle-income countries (LMICs), particularly Africa. The 68th United Nations World Health Assembly called for action to reduce the burden of OAP in LMICs [2]. Taking action to reduce exposure to OAP warrants the assessment of the exposure level through personal and environmental monitoring.

Several epidemiologic studies have found that ambient air pollution (from generators, vehicular emissions, agriculture and open burning, and household air pollution) is associated with risk of developing a broad range of diseases [3–5]. Ambient air pollution, for example particulate matter [6], is classified by the International Agency for Research on Cancer (IARC) as a group 1 carcinogen to humans lungs [7]. Sulphur dioxide (SO2), Nitrous oxide (NOX), carbon monoxide (CO) and low molecular weight particulate matter (e.g. PM_2.5_) from traffic-related air pollution (TRAP) are likely important contributors to OAP and have been extensively measured. Several potential biomarkers of OAP have been studied e.g., fraction of exhaled nitric oxide (FeNO), spirometry parameters, constituent cytokines, exhaled breath condensate, and induced sputum [8]; however, these measurements need technical expertise and may be expensive to carry out. In addition, they cannot be performed routinely in developing countries where the burden of OAP may be substantial and the majority of exposed urban citizens live.

Exhaled carbon monoxide (exhCO) has been used routinely and successfully for monitoring in the context of tobacco smoking cessation [9, 10]. Hence, it is conceivable that it could be used as an index of exposure to other air pollution sources other than tobacco smoking such as ambient air pollution which contains CO. CO rapidly combines with hemoglobin to form carboxyhemoglobin when inhaled; and its concentration in ambient air and duration of exposure are the most important determinants of carboxyhemoglobin saturation [11, 12]. The half-life of inhaled CO varies from 2 to 6 h depending on physiologic factors such as respiratory rate, making it a potential marker of recent exposure [13, 14]. Few studies have evaluated the correlation between exhCO with exposures from CO in solid fuels used in the households [15, 16], but none examined its relationships with OAP exposures. This study aimed to assess changes in exhCO over a shift of 8 h and its relationship with CO exposures following a short-term exposure to OAP in exposed males in Cotonou. Cotonou is the economical capital of Benin and has a quite elevated level of pollutants [17, 18]. We hypothesized that exhaled CO will increase over the shift and the post-shift exhaled CO will be associated with the mean value of CO measurement in ambient air of the last 2, 4 and 6 h of exposure.

## Methods

### Study design and population

This cross-sectional study included 170 male commercial motorcycle riders exposed to CO from OAP in Cotonou, the economical capital of Benin, an urban area with a lot of traffic, and hence high OAP.

The recruitment plan has been described extensively elsewhere [3]. Briefly we recruited 85 commercial motorcycle riders working in Cotonou and individually matched controls. The controls included civil servants and craftsmen mainly also working in Cotonou. All participants were asked not to smoke or be involved in cooking activities during the day of the measurement.

### Data collection and measurement

#### Questionnaire ﻿(see Additional file 1)﻿

We recorded socio-demographic data including age, marital status, educational level and daily income. In addition, we collected data on any physician-diagnosed medical condition such as; lung cancer, heart disease, tuberculosis, stroke, high blood pressure, diabetes, emphysema, asthma, chronic bronchitis or chronic obstructive pulmonary disease. Each co-morbidity was assessed individually but for this analysis, results were pooled and only the variable “any co-morbidity” was considered. Similarly we defined “any tobacco exposure” as ever smoking or exposure to environmental tobacco smoke. This has been pooled this way in order to put together all the source of CO from tobacco exposure other than ambient air pollution. We also collected data on their exposure to biomass (wood, coal, kerosene, crop residues) from cooking in their household.

#### Outdoor air pollution exposure

Carbon monoxide in ambient air (ambCO) was measured with a pre-calibrated portable CO data logger with a USB interface El-USB-CO® (Lascar Electronics, Whiteparish Salisbury, UK). All the participants carried the device for 8 h per day during working hours with a logging rate of 5 min. The device was hung around their neck before starting their working day and was removed 8 h later at the end of their shift.

#### Exhaled CO

exhCO was measured before and at the end of the shift using a CO Check + ® (MD Diagnostics Ltd., Maidstone, Kent, UK). The CO Check + ® is a portable device in which participants were asked to blow through a disposable mouthpiece for about 10 to 15 s. Two measurements were done, and if not identical a third one was repeated. The average of the three measurements was then reported. The device was calibrated using a 20 ppm CO in air calibration gas prior to all the measurements. The measurements were done in our local laboratory where the environmental CO was close to zero.

#### Spirometry

Spirometry was performed at the beginning of the study by each participant in the morning hours. The study participants completed spirometry according to the American Thoracic Society and European Respiratory Society guidelines [19] with an EasyOne® Spirometer (ndd, Switzerland) calibrated daily with a 3-L syringe. The spirometric procedure included at least 3 acceptable and repeatable forced vital capacity maneuvers. All the tests were reviewed by an external investigator and the best value was selected for analysis. We reported the abnormal lung function including obstructive and restrictive patterns.

### Data analysis

Individuals with incomplete records (technical problems from the devices handling, *n* = 17) were excluded from the analysis. Mean values of the initial and the end of the shift exhCO were compared using t - test. Mean values of ambCO for the last 2, 4 and 6 h before the measurement of the exhCO at the end of the working day were also compared using t-test. This range of time was used relative to the half life of CO which is 2 to 6 h depending on physiologic factors such as respiratory rate, making it a potential marker of recent exposure [13, 14]. A linear regression analysis was developed to assess the association between exhCO at the end of the shift and the ambCO for the last 2, 4 and 6 h of exposure. We adjusted for exposure to tobacco smoke, exposure to biomass, any co-morbidity, abnormal lung function and marital status. Regression coefficient was calculated. The statistical significance was set to 0.05. Analysis was done using Stata 12.

## Results

### Characteristics of the study participants

The mean ± SD age of our study population was 42.2 ± 8.4 years and they had a low income (Table 1). More than 90% were exposed to biomass smoke, and 32.5% reported exposure to tobacco smoke including 3 current smokers and 1 ex smoker.

**Table 1:** Characteristics of study participants

Characteristic	Summary
Age (years) - mean (SD)	42.2 (8.4)
Income per day ($)^a^ – mean (SD)	7.3(2.7)
Marital status n (%)	
Married	150(98)
Unmarried (single or widow)	3(2)
Education level n (%)	
None	22(14.4)
Primary school	75(49.1)
College	21(13.7)
High school	32(20.9)
University	3(1.9)
Biomass exposure n (%)	144(94.7)
Exposure to tobacco smoke n (%)	51(33.3)
Any co-morbidity n (%)	29(19)
Abnormal Lung function n (%)	23(15)

^a^1$ = 500XOF (Local currency in Benin)

### Exposures measurement

Mean ± SD exhCO increased from 13.4 ± 7.6 ppm (pre shift) to 18.5 ± 9.5 ppm (post shift) (*p* = 0.004) as shown in Table 2.

**Table 2:** Level of exhaled carbon monoxide and carbon monoxide in ambient air level

Variables	Mean	Standard deviation	p
Baseline exhCO (ppm)	13.4	7.6	
exhCO at the end of the working day (ppm)	18.5	9.5	0.004
2 last hours ambCO^a^	5.6	4.4	
4 last hours ambCO^a^	5.7	4.5	0.01
6 last hours ambCO^a^	6.3	4.7	
ambCO	6.6	3.8	

*ambCO* ambient carbon monoxide, *exhCO* exhaled carbon monoxide

^a^Mean values of ambCO before the measurement of exhCO at the end of the working day (in ppm)

### Association between exhaled CO at the end of the working day and CO in ambient air

Exhaled CO at the end of the shift was significantly associated to exposure to ambCO during the last 2, 4 and 6 h, even after adjusting for potential confounders. This association was stronger for ambCO measured over the most recent 2 h (regression coefficient β = 0.34) than for 4 and 6 h (regression coefficients of 0.22 and 0.14, respectively) (see Table 3).

**Table 3:** Exposure-response between exhaled CO at the end of the working day and CO in ambient air

	2 last hours AmbCO			4 last hours AmbCO			6 last hours AmbCO		
	β^a^	β^b^	p	β^a^	β^b^	p	β^a^	β^b^	p
exhCO at the end of the shift	0.31	0.34	< 0.001	0.20	0.22	< 0.001	0.13	0.14	0.01
Exposure to tobacco smoke		0.12	0.17		0.17	0.27		0.11	0.54
Exposure to biomass		0.07	0.08		0.09	0.13		0.19	0.10
Any comorbidities		0.11	0.14		0.12	0.14		0.09	0.14
Abnormal lung function		0.01	0.25		0.03	0.20		0.05	0.18
Marital status		0.02	0.28		0.01	0.21		0.04	0.16

*ambCO* ambient carbon monoxide, *exhCO* exhaled carbon monoxide

^a^crude values

^b^adjusted values

The difference between exhCO in the morning and the end of the working day (∆exhCO) was significantly associated to the mean of the 2 last hours ambCO (regression coefficient β =0.41, *p* = 0.008) and 4 last hours ambCO (regression coefficient β =0.21, *p* = 0.01) after adjustment on the same potential confounders as in Table 3.

## Discussion

We are not aware of other studies that have assessed the relationship between exhaled CO and short-term exposure to CO in ambient air from outdoor air pollution, especially traffic emissions. We have demonstrated that exhaled CO had a stronger association to 2-h ambient CO exposure (β = 0.34, *p* < 0.001) compared with the last 4 and 6 h of exposure. The weaker association of the exhaled CO to the average values of the last 4 and 6 h CO exposure may be due to the half-life of the inhaled CO. The half-life of CO is 2–6 h and it is thus understandable that exhCO might better reflect the most recent 2-h exposure. Moreover, we found a significant difference between the measurements of baseline exhCO and exhCO at the end of the 8-h shift. This difference was strongly associated with the average values of the 2 last hours of exposure to CO from outdoor air pollution. The baseline exhaled CO was 13.4 ± 7.6 ppm and this is higher than the cut-off value of 6 to 9 ppm commonly used to distinguish smokers from non-smokers [14, 20]. Although the baseline elevation in exhaled CO could be related to other causes, it is most likely related to the ambient exposure the study participants were exposed to before they arrived at our local laboratory in the morning hours. This high baseline exhCO compared to the cut-off value used in evaluating the success of smoking cessation, and the pre-post work shift difference supports the clinical relevance of air pollution exposure for commercial motorcycle riders and other groups who work on and/or live near roads with heavy traffic.

This study also showed that measurement of exhaled CO is feasible in a limited resource setting. The level of carboxyhemoglobin can be calculated from the Coburn-Forster-Kane Equation. We were able to measure the baseline exhaled CO and the exhaled CO after 8 h in individuals exposed to OAP. Indeed, study participants were asked to come into our local laboratory in Benin for the measurement. This can be done routinely in a health care center with a relatively inexpensive device and low-cost disposable mouthpieces. In a patient for whom the healthcare provider suspected that exposure to OAP is a risk factor, exhCO measurement can be done to confirm exposure or for assessing intervention effectiveness. A cost-effectiveness analysis is needed to confirm whether this simple and non-invasive biomarker of OAP exposure is indeed useful in patient care.

We assumed that all the exposures happened outdoors based on the participant’s declaration and the hours of measurements although we did not use a device like a GPS tracker to confirm it.

## Conclusion

In non-smoking and non-cooking men exposed to OAP, exhCO can be used as a biomarker of short-term exposure to ambCO. Therefore, exhCO can be used to assess exposure to air pollution in less wealthy countries where this test is already routinely used in evaluating smoking cessation programs.

## Additional file

Additional file 1: Questionnaire. (DOCX 11 kb)

## Abbreviations

AmbCO: Ambient carbon monoxide
CO: Carbon monoxide
ExhCO: Exhaled carbon monoxide
OAP: Outdoor air pollution
ppm: Part per million
SD: Standard deviation
