# Supplementary material for: Exhaled carbon monoxide: a non-invasive biomarker of short-term exposure to outdoor air pollution
Source: BMC Public Health. 2017 Apr 17;17:320. doi: 10.1186/s12889-017-4243-6 (PMC5392985; doi:10.1186/s12889-017-4243-6)
Supplement: Additional file 1: — Questionnaire. (DOCX 11 kb) [file 12889_2017_4243_MOESM1_ESM.docx]

**Questionnaire**

What is your date of birth? ____ ____/____ ____/____ ____ ____ ____

d d m m y y y y

Marital status:  married  Widow single

Educational level:  None  Primary school  College

 High school University

What is your daily Income? : ………… XOF

Has a doctor or other health care provider ever told you that you have:

 lung cancer  heart disease  tuberculosis  stroke  high blood pressure  diabetes  emphysema  asthma  chronic bronchitis  chronic obstructive pulmonary disease

Have you ever smoked? :  Yes  No

Have you been passively exposed to tobacco smoke? :  Yes  No

Are you exposed to biomass (wood, coal, kerosene, crop residues) when cooking in your household for at least 6 months in your life time? :  Yes  No
